# Toward Sustainable Adsorbents for CO_2_ Capture: Reuse of Silica from Spent Silica-Polyethylenimine

**DOI:** 10.1021/acs.energyfuels.6c00149

**Published:** 2026-04-03

**Authors:** Wei Li, Lee Stevens, Stylianos D Stefanidis, Angelos Lappas, Daniele Fabbri, Laura Mazzocchetti, Irene Coralli, Simon Stebbing, Colin E. Snape

**Affiliations:** a Low Carbon Energy and Resources Technologies Group, Faculty of Engineering, 6123 University of Nottingham, Nottingham NG7 2TU, U.K.; b Chemical Process and Energy Resources Institute, Centre for Research and Technology-Hellas (CERTH), Thessaloniki 57001, Greece; c Department of Chemistry Giacomo Ciamician, University of Bologna, Technopole of Rimini, via Dario Campana 71, Rimini, Bologna 40126, Italy; d Department of Industrial Chemistry Toso Montanari, 9296 University of Bologna, via Gobetti 85, Bologna 40136, Italy; e PQ Corporation, Bank Quay, Liverpool Road, Warrington WA5 1AQ, U.K.

## Abstract

Silica-polyethylenimine (silica-PEI) and, more recently, silica-alkoxylated polyethylenimine (APEI) adsorbents are promising materials for CO_2_ capture due to their high adsorption capacity and selectivity. The ability to reuse silica supports recovered from spent adsorbents can enhance overall material efficiency and facilitate the development of regeneration strategies given our earlier work demonstrating that spent PEI generates pyrazines and other chemicals during pyrolysis. In this study, spent silica-APEI adsorbents were subjected to four different treatments to recover silica supports and to investigate how thermal regeneration influences pore structure and subsequent reimpregnation performance. Single-stage and two-stage pyrolysis at 500–600 °C resulted in moderate mesopore contraction (13%) and total pore volume reduction (10%) compared to the initial silica, largely independent of the specific pyrolysis route applied. The recovered silicas were successfully reimpregnated with fresh PEI, exhibiting an optimal loading of 45 wt %, slightly lower than that of the original silica (47 wt %). At optimal PEI loadings, the regenerated silica-PEI displayed an approximately 10% reduction in CO_2_ adsorption capacity relative to fresh silica-based adsorbents. These results demonstrate that silica supports largely retain functional compatibility with PEI after high-temperature pyrolysis, laying the foundation for further studies with multiple cycles to assess the overall cost and life cycle benefits.

## Graphical abstract

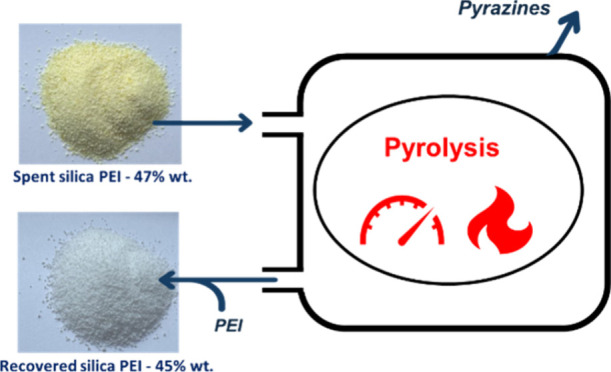

## 1 Introduction

Rapid deployment of carbon capture and storage (CCS) is essential to limiting global warming and mitigating the impacts of climate change.^1–6^ Meeting global net-zero targets by 2050 will require the removal of up to 10 Gt CO_2_ per year.^7^ Among the available capture strategies, postcombustion CO_2_ capture (PCC) offers an immediate and scalable route because it can be integrated into existing energy and industrial infrastructure.^1–4,8,9^ Meanwhile, direct air capture (DAC)^10–12^ has emerged as a key technology due to its ability to remove CO_2_ directly from the air, offering a highly flexible solution.^11,13–15^ Scaling PCC and DAC to the levels required for climate mitigation demands sorbents that combine high performance with cost-effectiveness and full lifecycle sustainability.^16–18^

Mesoporous silica-supported polyethylenimine (PEI), and more recently silica-alkoxylated polyethylenimine (APEI),^19–21^ has emerged as one of the most promising classes of solid amine adsorbents for PCC and DAC,^20,22–26^ due to their high CO_2_ uptake at a wide range of adsorption temperatures (25–70 °C), strong CO_2_ selectivity at low partial pressures, and relatively mild regeneration conditions.^20,27–29^ The mesoporous structure effectively supports dispersing PEI/APEI, thereby increasing their available contact surface area and enhancing CO_2_ adsorption capacity. Recently, a fully organic solvent-free synthesis route for silica-PEI and silica-APEI has been developed,^15^ offering significantly reduced production costs. In addition, the successful scale-up of testing from laboratory setups to hundreds of kilograms demonstrates the potential of silica-PEI systems for large-scale CO_2_ capture,^22^ bringing this technology closer to real-world applications. However, most prior research on silica-PEI and related amine-functionalized adsorbents has focused on improving initial CO_2_ adsorption performance,^21,30^ enhancing oxidative and thermal stability,^21,30,31^ or/and determining how many adsorption-regeneration cycles fresh materials can withstand during their active lifetime.^21,32^ In contrast, very little attention has been given to what happens once these sorbents reach the end of their operational life, leaving their fate, recyclability, and reuse potential largely unexplored, posing a major barrier to industrial deployment and accurate sustainability assessment.

To develop circular end-of-life strategies of silica-PEI/APEI sorbents, recently, we have used pyrolysis to recover potentially valuable chemicals from spent PEI while simultaneously enabling the recovery of the silica support.^33–35^ The useful chemicals from the pyrolysis of PEI include linear aliphatic amines, piperazines, and pyrazines, with potential applications, particularly in the food industry.^33^ Qualitatively, the same chemicals were generated from pyrolysis of silica-PEIs, extracted PEIs from silica, and the residual PEI in silica postextraction, regardless of the oxidation degree of the materials. Notably, pyrazines are the predominant pyrolysis products in the pyrolysates from pre- and postextracted silica-PEI, formed by alkylation and aromatization reactions.^33,34^ More importantly, pyrolysis also yields a solid silica residue, indicating the potential to reuse the recovered silica as a support for PEI. However, the structural evolution of the recovered silica; the extent to which its porosity is retained; and critically, its suitability for reimpregnation and reuse need to be evaluated.

We investigate here how different recovery processes influence the structural integrity of the regenerated silica, particularly mesopore volume and overall porosity, and assess whether the recovered silica can be successfully reimpregnated with PEIs and maintain CO_2_ adsorption performance comparable to the initial silica-PEI materials. This is achieved by directly comparing the textural properties and CO_2_ uptake of regenerated silica-PEIs with those of fresh materials.

This study offers an initial experimental demonstration of the technical feasibility of recovering silica from fully spent silica-APEI adsorbents and reusing it as a functional support for PEI reimpregnation. The results establish a baseline for evaluating how high-temperature thermal treatments affect silica structure and performance while highlighting the need for further studies to address multicycle regeneration, alternative recovery routes, and more detailed process-level analyses.

## 2 Experimental Section

### 2.1 Material Preparation

The initial silica was obtained from PQ Corporation and prepared according to the production methods described by Iler and Brinker and Scherer.^36,37^ This silica was impregnated with two commercial PEIs obtained from BASF SE, FG (LUPASOL FG, Mn ∼800 g/mol) and G100 (LUPASOL G100, Mn ∼5000 g/mol), at loadings of 30–50 wt %, as well as with an APEI, FG-0.22PO (Mn ∼1040 g/mol), in which propylene oxide is used to alkoxylate 22% of the primary and secondary amines,^15^ at a loading of 47 wt %. The recovered silicas obtained after pyrolysis from oxidative degradation were also impregnated with FG and G100 (30–50 wt %) to determine the optimal loading for each silica type. The impregnation was carried out using a minimal amount of water, following the procedure detailed in our previous work.^15^ The resulting silica-PEI materials were then tested for CO_2_ adsorption and desorption to evaluate their adsorption performance and regeneration conditions, as detailed in Section 2.4.

To simulate actual operating conditions in which oxidation occurs during repeated adsorption-regeneration cycles, the silica-APEI sample (5 kg) was subjected to oxidative degradation in a fluidized bed reactor (10 L/min air, 1 bar) at 60 °C for 60 days. Under these conditions, the CO_2_ adsorption capacity at 50 °C decreased by 43%, from 10.6 to 6.1 wt %.^15^ These oxidized silica-PEI samples represent the spent adsorbents after extended use (Figure 1). Subsequently, four different pyrolysis procedures were applied to the spent silica-APEI to obtain recovered silica, as detailed in Section 2.3. Finally, the recovered silicas were reimpregnated with fresh PEI (FG and G100) to produce regenerated silica-PEI materials, and their CO_2_ adsorption performance was compared with the initial silica-PEI samples.

**Figure 1:**
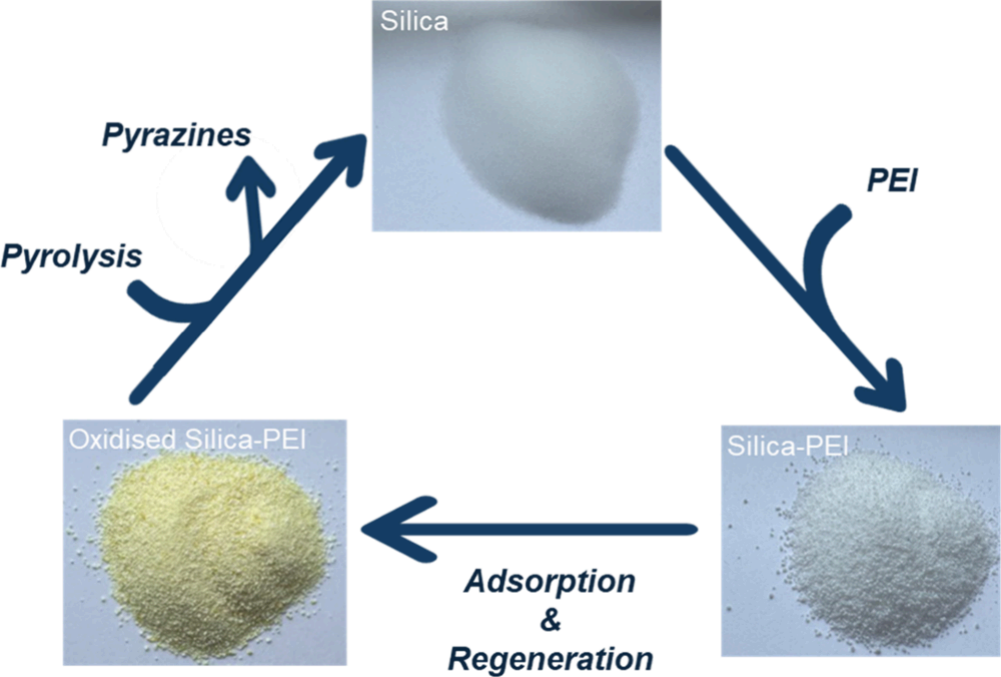
Process cycle of silica-PEI preparation, oxidation, and pyrolysis.

### 2.2 Determination of Pore Textural Properties

The pore textural properties of the initial silica and recovered silicas were characterized using a Micromeritics ASAP 2420 surface area and porosity analyzer (Micromeritics Instrument Corp., USA). Approximately 350 mg of each sample was placed into a sample tube fitted with a filler rod. Before analysis, samples were degassed under high vacuum (<0.013 mbar) to eliminate adsorbed moisture and gases. Degassing was conducted at 250 °C for 15 h for the initial and recovered silicas.

Nitrogen sorption isotherms were collected over a relative pressure (P/Po) range of 0.01–0.99 at −196 °C. Specific surface areas were calculated using the Brunauer–Emmett–Teller (BET) model in the relative pressure range of 0.05–0.20, ensuring a positive BET “C” constant.^38^ The volume of mesopores (pore width 2–50 nm) and macropores (pore widths from 50 to 140 nm) and pore size distributions were determined from the adsorption isotherms using the Barrett–Joyner–Halenda (BJH) method with Broekhoff-de Boer thickness correction. Micropore (pore width <2 nm) volumes were estimated using the Dubinin–Radushkevich (D-R) model.^38–40^

### 2.3 Pyrolysis of Spent Silica-PEI

The spent silica-PEIs used for pyrolysis have a 47 wt % loading of APEI (FG-0.22PO); this optimal loading is consistent for the APEI and two PEIs used.^15^ A pyrolysis temperature of 500–600 °C was selected to ensure complete PEI removal while preserving the silica structure and textural properties.^33–35^

Approximately 50 g of spent silica-APEI was subjected to pyrolysis in a stainless-steel fixed-bed reactor operated at 1 bar. The recovered silicas, denoted RS-500 and RS-600, were produced by single-stage pyrolysis at 500 and 600 °C, respectively; in each case, the reactor was heated to the target temperature and maintained isothermally for 340 min under a continuous nitrogen flow (55 mL/min). Recovered silicas, denoted RS-2SN and RS-2S, were also obtained by a two-stage pyrolysis treatment. In the first stage, the material was heated to 150 °C for 30 min under nitrogen flow (45 mL/min) to remove water. In the second stage, the temperature was increased to 500 °C and maintained for 120 min, either under nitrogen flow (RS-2SN) or without flow (RS-2S). All the recovered silicas were subsequently calcined in air at the corresponding pyrolysis temperature (500 or 600 °C) for 120 min.

Furthermore, the initial silica (approximately 10 g) was placed in a horizontal tube furnace and heated from ambient temperature to either 500 or 600 °C in air (1 L/min, 1 bar). The samples were held at each temperature for 180 min to obtain two blank silica samples for comparison, which were denoted Silica-500 and Silica-600, respectively.

### 2.4 CO_2_ Adsorption Capacity

The CO_2_ adsorption capacities of silica-PEI samples with varying PEI loadings (30–50 wt %) were determined using thermogravimetric analysis (TGA). Approximately 25 mg of each sample was placed in the TGA pan. To remove adsorbed moisture, samples were pretreated by heating to 110 °C for 30 min under a nitrogen flow (1 bar, 100 mL/min). After that, the temperature was reduced to 75 °C, which is identified as the optimal adsorption temperature for the PEI-FG and G100 for CO_2_ adsorption, since at 75 °C, both the CO_2_ adsorption capacity and adsorption kinetics were highest compared with those measured at other temperatures (25–120 °C).^15^ Subsequently, the gas stream was switched to a mixture of 15% CO_2_ in nitrogen (1 bar, 100 mL/min), with the samples held isothermally at 75 °C for 60 min to ensure equilibrium adsorption. The CO_2_ uptake was determined based on the weight change during exposure to the CO_2_/N_2_ gas mixture and reported on a dry basis.

### 2.5 Scanning Electron Microscopy (SEM)

Cross-sectional imaging and elemental analysis were carried out using a Zeiss Crossbeam 550 focused ion beam-scanning electron microscope (FIB–SEM). Samples were mounted on aluminum stubs with conductive carbon cement and cured for 48 h before analysis. Fracturing and site-specific cross-sectioning were performed with the FIB to expose the internal microstructure. The exposed surfaces were sputter-coated with platinum to reduce charging and enhance image quality. Energy-dispersive X-ray spectroscopy (EDS) was conducted in the SEM chamber to map elemental distributions, with particular attention to nitrogen (N) and silicon (Si).

## 3 Results and Discussion

### 3.1 Properties of Initial and Recovered Silicas

The BET surface areas with the pore volumes and the pore size distributions of recovered silicas and the initial silica are summarized in Table 1 and Figure 2, respectively. Compared to the initial silica (surface area of 306 m^2^/g, micro, meso and total pore volume are 0.114, 1.60, and 1.72 cm^3^/g), all the recovered silica samples, RS-500, RS-600, RS-2S, and RS-2SN, display higher BET surface area (330, 326, 316, and 321 m^2^/g), slightly increased micropore volume (0.121, 0.117, 0.114, and 0.115 cm^3^/g), and reduced mesopore (1.42, 1.36, 1.39, and 1.38 cm^3^/g) and total pore volumes (1.55, 1.49, 1.51, and 1.50 cm^3^/g, Table 1), respectively. Minor variations in pore texture are observed among the recovered silica from different pyrolysis methods (Table 1).

**Table 1:** Pore Texture Properties of the Initial and Recovered Silica Samples, including BET Surface Area, Micropore, Mesopore, and Total Pore Volumes

Sample	BET SA (m^2^/g)	*V*_micro_ (cm^3^/g)	*V*_meso_ (cm^3^/g)	*V*_tot_ (cm^3^/g)
initial silica	306	0.114	1.60	1.72
Silica-500	310	0.115	1.62	1.74
Silica-600	311	0.115	1.62	1.74
RS-500	330	0.121	1.42	1.55
RS-600	326	0.117	1.36	1.49
RS-2S	316	0.114	1.39	1.51
RS-2SN	321	0.115	1.38	1.50

**Figure 2:**
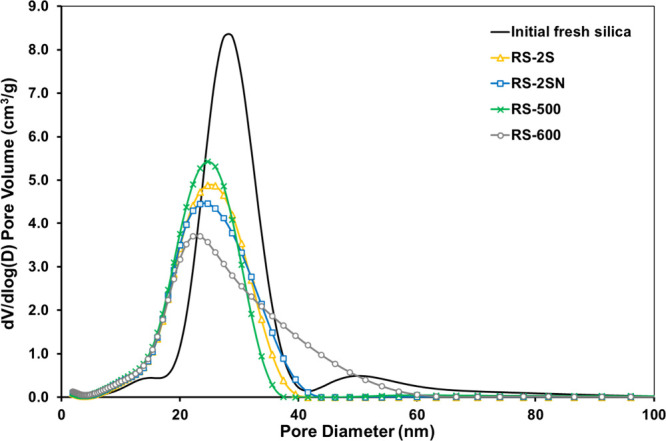
Pore size distribution of the initial and recovered silicas up to a pore size of 100 nm.

The pore size distribution up to 100 nm (Figure 2) shows the differences between the initial and recovered silica samples. The initial silica exhibits two main peaks: a dominant one centered at about 28 nm (range: 20–40 nm) and a smaller peak around 50 nm (range: 40–60 nm). In contrast, all recovered silica samples display a single main peak shifted to below 24 nm. RS-500, RS-2S, and RS-2SN show peak ranges from 15 to 40 nm, while RS-600 presents a broader distribution from 15 to <60 nm. This shift likely results from the collapse of larger mesopores into smaller ones during pyrolysis, driven by PEI decomposition and then partial framework shrinkage. This interpretation is consistent with the control experiment, in which the pore volumes of Silica-500 and Silica-600 remained essentially unchanged and are comparable to the initial silica, after exposure to high-temperature air (500 and 600 °C for 180 min) (Table 1). These results indicate that the observed changes in pore size distribution and mesopore collapse are primarily associated with PEI decomposition and volatilization.

Although some structural pore collapse has occurred in the recovered silicas, changes remain primarily in the mesopore range, but some smaller pores may shift toward the micropore range. This explains the slight increase in BET surface area and micropore volume in the recovered samples. Mesopore volume loss is minimal, approximately 13% compared to the initial silica. Similarly, the total pore volume of the recovered silica decreases by around 10%, likely attributable not only to partial pore collapse but also to any residual materials blocking the pore.

From a thermal processing perspective, the observed mesopore reduction is consistent with partial framework densification induced by polymer decomposition and the removal of pore-filling organic species during pyrolysis. The collapse of larger mesopores into smaller mesopore likely reflects localized stress release within the silica network as PEI volatilizes. Importantly, the absence of significant pore volume loss in the thermally treated blank silica confirms that the structural changes are driven primarily by PEI decomposition rather than by intrinsic instability of the silica framework.

### 3.2 Optimal Loading for the Initial and the Spent Silicas

The optimal PEI loading giving the maximum CO_2_ adsorption capacity for the initial silica was 47 wt.% within the tested range of 30–50 wt.% for both the FG and G100.^15^ Figure 3 shows that all four recovered silicas exhibit the same optimal loading of 45 wt.% for both PEIs (FG and G100), which is 2 wt.% lower than that of the initial silica (47 wt.%). The observed reduction of the optimal PEI loading for the regenerated silica can be mechanistically interpreted in terms of subtle but systematic changes in mesopore morphology. Although the reduction in mesopore volume is modest (13%), the consistency of the optimal loading across all regenerated silicas suggests that the sorbent’s capacity is principally governed by available mesopore volume^27,41,42^ rather than subtle differences in framework chemistry introduced during pyrolysis. In other words, once the mesopore volume drops from 1.60 cm^3^/g for the initial silica to approximately 1.40 cm^3^/g for the recovered samples, the material reaches a new equilibrium condition, in which 45 wt.% PEI represents the maximum amount that can be effectively accommodated without excessive pore blocking or diffusion limitations.

**Figure 3:**
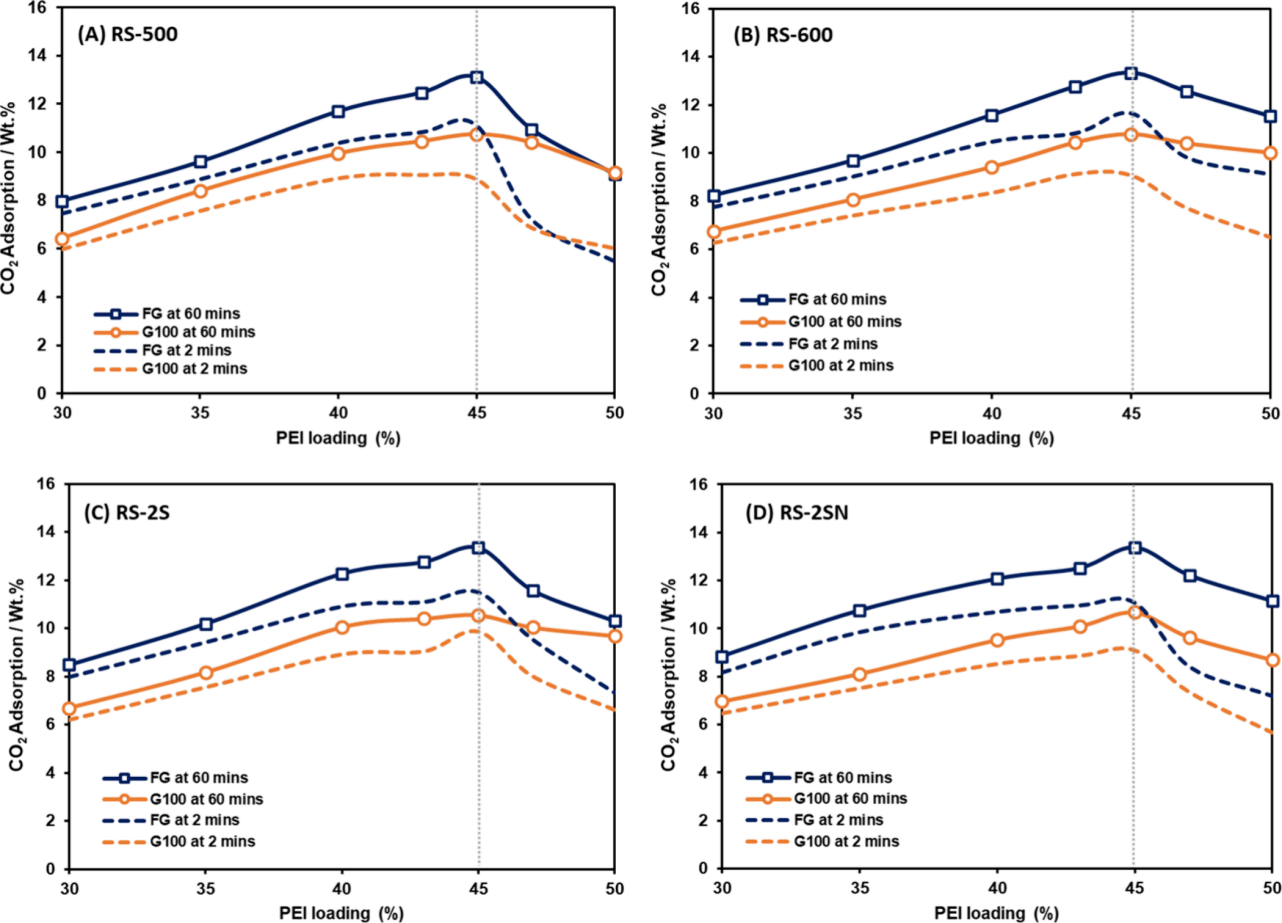
CO_2_ adsorption capacities at 2 and 60 min for recovered silica-PEIs with PEI loadings ranging from 30 to 50 wt.% at 75 °C under 15% CO_2_. (A–D) Silica samples RS-500, RS-600, RS-2S, and RS-2SN, respectively. The uptake at 60 min represents the equilibrium CO_2_ adsorption capacity, while the uptake at 2 min reflects the adsorption kinetics.^15^

Importantly, all regeneration methods, single-step, high- and low-temperature, and two-stage pyrolysis, produce nearly identical mesopore volumes and thus the same PEI optimal loading, indicating that the key structural change of recovered silicas is simply mesopore contraction rather than any method-specific chemical modification.

The very small differences in mesopore volume among regenerated samples (1.36–1.42 cm^3^/g) also explain why the optimal loading remains unchanged: variations of this magnitude are insufficient to meaningfully impact PEI accommodation. This consistency suggests that the silica framework is structurally robust and that PEI loading behavior is largely governed by mesopore volume, making the regeneration process highly reproducible and reliable for long-term use.

### 3.3 CO_2_ Adsorption Performance

At the same PEI loading of 45 wt.%, the recovered and initial silica exhibit comparable CO_2_ adsorption performance (Figure 4). For the recovered silica-PEIs, the CO_2_ uptake is close to 13.3 wt.% for FG (13.1, 13.3, 13.3, and 13.4 wt.% for RS-500, RS-600, RS-2S, and RS-2SN, respectively) and around 10.7 wt.% for G100 (10.8, 10.8, 10.6, and 10.7 wt.%). These values closely match those of the initial silica-PEI samples at 45 wt.% PEI (13.3 wt.% for FG and 10.9 wt.% for G100) and are slightly lower (c*a*. One wt.%) than those at 47 wt.% PEI (14.2 wt.% for FG and 11.8 wt.% for G100).

**Figure 4:**
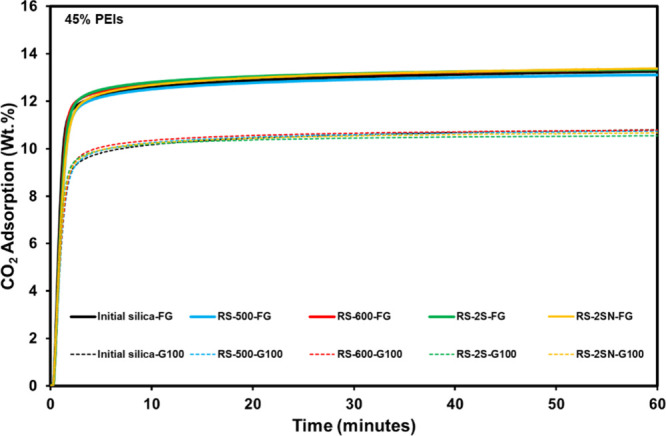
CO_2_ adsorption isotherms of initial and recovered silica-PEIs (FG and G100) at 45 wt.% at 75 °C under 15% CO_2_. Solid lines represent FG, and dashed lines represent G100.

These results highlight the following important facts:

First, the PEI content is the dominant factor controlling CO_2_ adsorption. When the same amount of PEI is used, the CO_2_ uptake remains essentially the same, regardless of whether the support is the initial or recovered silica. Second, the fact that all the recovered silicas display indistinguishable CO_2_ uptakes indicates that PEI infiltrates the regenerated mesopore network in the same manner as in the fresh silica.

Furthermore, the scanning electron microscopy (SEM) and energy-dispersive X-ray spectroscopy (EDS) images of nitrogen (N) and silicon (Si) in the cross sections of the initial silica-APEI (Figure 5A) and the recovered silica (RS-600)-FG (Figure 5B), both at 45 wt.%, confirm that PEI impregnation occurs within the internal pores of the silica rather than only on the surface. This is evident from the uniform distribution of N across the observed area in the EDS mapping (Figure 5A), where N originates from PEI and Si is present only in the silica. Similar results are observed for the recovered silica-PEI, indicating that the recovered silica obtained after pyrolysis retains a pore structure suitable for a new cycle of PEI impregnation (Figure 5B). These findings demonstrate that the recovered silica continues to function effectively in dispersing PEI within its porous framework.

**Figure 5:**
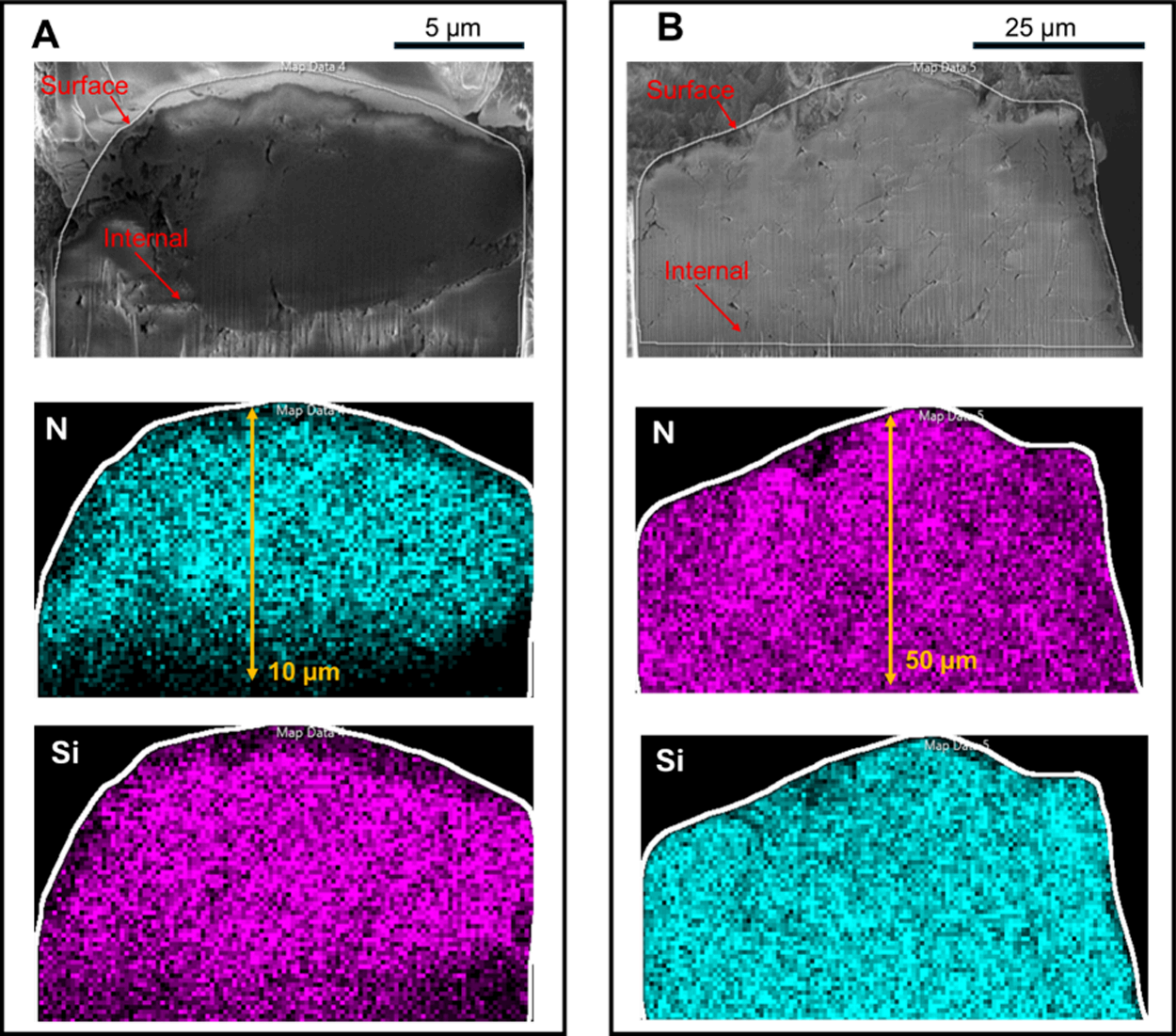
SEM and EDS elemental maps (nitrogen-N; silicon-Si) of cross sections of (A) initial silica-APEI and (B) recovered silica (RS-600)-FG, both at 45 wt.%.

## 4 Conclusions

This study provides the first experimental evidence that silica supports recovered via high-temperature pyrolysis can be successfully reimpregnated with PEI, while retaining functional CO_2_ adsorption performance and gaining the economic benefits from pyrazines and other products generated by PEI decomposition. Pyrolysis-based regeneration preserved up to approximately 90% of the original pore volume, enabling an optimal PEI loading of 45 wt %, only slightly lower than that of the initial silica (47 wt %). At identical PEI loadings, the regenerated silica-PEI materials exhibited CO_2_ adsorption capacities and kinetics comparable to those of the corresponding fresh silica-based adsorbents.

Across all pyrolysis routes investigated, including single-stage and two-stage treatments at 500–600 °C, the recovered silicas showed minimal variation in textural properties, with a consistent optimal PEI loading (45 wt %) and a modest mesopore reduction of approximately 13%. The observed CO_2_ adsorption performance reduction of about 10% relative to the initial materials is primarily attributed to changes in mesopore size distribution rather than to fundamental degradation of the silica framework. Overall, the results demonstrate the thermal robustness of silica supports under pyrolysis conditions and establish a baseline for evaluating the effects of thermal regeneration on porous silica-based adsorbents. Beyond this initial demonstration, the findings suggest that the thermal regeneration of silica supports may contribute to improved material utilization, although a comprehensive assessment of sustainability, cost, and scalability remains the subject of future work.
